# Depressive symptoms and negative life events: What psycho-social factors protect or harm left-behind children in China?

**DOI:** 10.1186/s12888-017-1554-1

**Published:** 2017-12-15

**Authors:** Yu Guang, Zhengzhi Feng, Guoyu Yang, Yaling Yang, Lifei Wang, Qin Dai, Chaobing Hu, Keyu Liu, Rui Zhang, Fan Xia, Mengxue Zhao

**Affiliations:** 1 School of Psychology, Third Military Medical University, Chongqing, 400038 China; 2 Chongqing Foundation for Poverty Alleviation, Chongqing, 400020 China; 3 Department of Nursing Psychology, School of Nursing, Third Military Medical University, Chongqing, 400038 China; 4 Key Applied Psychology Lab, Chongqing Normal University, Chongqing, 401331 China

**Keywords:** China, Left-behind children, Depressive symptom, Negative life event, Mental health, Migrant parent, Communication

## Abstract

**Background:**

In China, children under 18 years old who are left at rural residences for at least 6 months by either one or both of their parents migrating to work in cities are called “left-behind children (LBC)”. Due to restricted family support, they are at a greater risk of developing depressive symptoms than non-left-behind children (NLBC). The objective of this study is to explore how depressive symptoms and stress induced by negative life events such as interpersonal conflicts, punishment and loss, as well as their relationships vary for LBC with different left-behind-related characteristics.

**Methods:**

Using data from a large school-based survey conducted in Chongqing between December 2012 and June 2013, we first identified the differences in depressive symptoms and negative-event-induced stress between LBC and NLBC, and then analyzed the variances among LBC with different left-behind-related characteristics. The data was analyzed with Chi-square test, MANCOVA, ANCOVA, ANOVA, T-test and hierarchical multiple regression analyses.

**Results:**

We found that LBC were more stressed when experiencing negative events and had more depressive symptoms than NLBC. Children left behind by both parents were most depressed. Negative-event-induced stress and communication on life difficulties with migrant parents were risk factors for depressive symptoms, whereas adequate communication on academic performance or children’s feelings was a protective factor against depressive symptoms. Communication duration and frequency, communication by visiting, communication on academic performance, life difficulties and children’s feelings moderated the relationship between stress and depressive symptoms, respectively. Duration of separation, communication duration and frequency, communication on academic performance, learning difficulties and children’s feelings moderated the relation between the type of parental migration and depressive symptoms, respectively.

**Conclusions:**

Our findings suggest that children left behind by both parents should be the focus of public attention for their higher susceptibility to stress-related depression. To help LBC stay mentally healthy, governments need to formulate regulations contributing to LBC’s family reunion, communities need to involve more residents to attend LBC as “surrogate parents” and teach migrant parents to communicate with LBC properly, and schools need to teach LBC how to deal with stress and communicate with migrant parents.

## Background

Left-behind children (LBC) are prevalent in modern China. These children who are under 18 years old are left at rural residences for at least 6 months by either one or both of parents migrating to work in cities, whereas their age-matched peers living with both parents at rural residences are called “non-left-behind children (NLBC)” [1].

In the past 30 years, the “Reform and Opening Up” policy has made some Chinese cities prosperous. However, people in areas where economic growth lags much behind are still struggling with poverty. To help families get rid of adverse circumstances, many young adults leave rural residences for cities in pursuit of better salaries, leaving their children at home. According to the result of the last national census of population [2], the number of LBC has reached over 60 million in China, occupying 21.88% of Chinese children. This special group not only distributes in underdeveloped areas of China, but also exists in developed regions such as Jiangsu, Guangdong and Shandong Provinces. Long-term separation from parents is common for LBC, which is partly caused by Chinese Hukou system which limits migrant population’s welfare at new residences. Such separation has been proved to associate with psychopathology [3], delinquency [4], and impaired cognition [5]. LBC have also been reported to experience more negative life events than NLBC [6].Therefore, it is urgent to better understand LBC’s psychological dynamics, especially considering the relationships between mental illnesses such as depression and stress induced by general negative life events like interpersonal conflicts, punishment and loss.

Recent studies have shown that LBC’s living environments are more stressed than those of NLBC [6–12]. Compared to NLBC, LBC have smaller social networks, lower life satisfaction, poorer nutritional status and more housework [7], and even take on a burden of attending aged or sick caregivers [8]. LBC are generally problematically reared and receive inadequate academic guidance from teachers and caregivers [13]. They also experience more negative life events [6] and feel a higher degree of strain and pressure derived from negative events such as interpersonal conflicts, academic pressure, punishment, loss and health problems [6, 9–11].

Furthermore, LBC in China are at a greater risk of depression [14–16]. It was previously reported that the likelihood of depression of LBC primary students in Grades 4–6 is two-three times higher than that of NLBC; the depression is linked to lower levels of social support and types of parental migration [14]. Wang et al. also found a higher likelihood of depressive symptoms in left-behind children (OR = 1.110, *p* < 0.05) [15]. Fan et al.’s study revealed that the greater risk of depression in LBC is linked to inadequate teacher support and the care of young caregivers or nonrelatives with poor education or low socioeconomic status [17].

Depression may follow any undesirable events that are significant for an individual [18–22].George Brown et al. carried out the most extensive studies documenting that women with major depression had an approximately 3-fold more likelihood of having a severely negative life event before onset than controls [23–26]. Kendler et al. further illustrated the causal role of life events in a 5-year study on dizygotic and monozygotic female-female twin pairs [27, 28]. Additionally, certain classes of events are especially likely to provoke depression. A line of research has demonstrated that people who lose their loved ones (e.g., death of family members) have a greater risk of developing depression [20, 29, 30]. Specifically, their anhedonia is elevated after deaths and romantic losses [20]. Interpersonal conflict is another factor contributing to depression [31–35], especially for raising the level of guilt [20]. Punishment increases the depression risk among youths, adolescents and children as well [36–41], which is moderated by parent support and gender of the supportive parent [41].Academic pressure [42–44] and health and adaptation problems [15, 20] have also been proved as main causes of depression.

## Theoretical framework

Some evidences pertaining to children’s psychological health indicate that children who are under stress or have experienced stressful life events are at a greater risk of developing major depression [45–48]. For example, a history of physical abuse, sexual abuse or neglect is associated with a 2- to 5-fold increase in the likelihood of depression among children, even after controlling for demographic and family contextual factors known to be related to depression onset [48]. Although these studies do not particularly focus on LBC, it is reasonable to infer that LBC may be more susceptible to negative-life-event-provoked depression. Cognitive models of depression [49, 50] and attachment theories [51] provide a theoretical framework for this hypothesis.

Cognitive models of depression share the view that maladaptive thoughts and negative appraisal of life circumstances lead to the onset and development of depression [49, 50, 52, 53]. The original theory by Aaron Beck [49, 50] uses schemas, the basic components of cognitive organization facilitating information process, to explain the pathogeny of depression: individuals with depressive self-schemas tend to attend, encode and retrieve schema-consistent information with bias, and to ignore or misinterpret positive and neutral information; and the depressive self-schemas are presumed to develop early in life but remain silent until they are triggered by adverse circumstances [49]. Therefore, people with depressive self-schemas are more likely to suffer from depression if they experience negative life events.

Attachment theories explain how the depressive self-schemas form. Early attachment experiences with primary caregivers influence children’s understanding of others’ reactions or attitudes toward their needs and performances. If children’s needs are inconsistently satisfied by caregivers in time, they incline to consider the world as insecure and themselves as abandoned, which consequently constitutes their depressive self-schemas. LBC are generally looked after by one parent, grandparents, older siblings and other relatives [54], who are usually incapable of fully satisfying LBC’s needs because of inadequate energy and experience [8]. Thus, compared to NLBC, LBC are more likely to form depressive self-schemas and develop depression.

The differences in paternal and maternal parenting may moderate the relationships between negative life events and depressive symptoms: fathers incline to play a role in setting discipline [55] and offering material support [56], while mothers play a prominent role in providing affection and caring [55, 56]. Main and Weston found that the security of infant-mother attachment is more strongly associated with the infants’ responses to an unfamiliar person than the security of infant-father attachment [57]. Cole and MecPherson found that father-child conflict but not mother-child conflict is positively related to depressive symptoms of adolescents [58]. A 6-year longitudinal study by Brody et al. shows that only paternal warmth has a significant long-term effect on shaping adolescents’ attitudes toward social issues such as sex roles, child support, welfare, marriage, divorce and teenage childbearing [59]. Finally, because of the general maltreatment from non-parent caregivers [6, 9–11], children left by both parents might experience more depressive symptoms and stress than other types of LBC. Hence, it is important to examine the effects of the type of parental migration on LBC’s psychology separately for their potentially varied outcomes.

Besides the type of parental migration, parent-child communication is another potential moderator for two reasons. First, children value themselves partially based on how others consider themselves [60]. By interacting with parents, children obtain information for constructing their self-schemas. Persistent negative feedback from parents increases the likelihood of forming negative self-schemas which further increases their vulnerability to depression [60]. Second, proper communication with parents enables children to learn stress manipulation skills. Accordingly, parent-child communication might influence the degree of stress and depressive emotion through shaping children’s self-regard and stress coping abilities. Demographic variables such as age, gender, ethnicity, single child in family and family incomes, and some left-behind-related variables such as age at separation and duration of separation are other potential moderators. This is because individuals who differ in these variables possibly vary in cognitive and genetic vulnerability to stress.

Following along these points, present investigation aimed to understand how the stress induced by negative life events and depressive symptoms vary for different groups of LBC and NLBC. We hypothesized that, in line with previous research, LBC would have more depressive symptoms and stress induced by negative events than NLBC. We also hypothesized that depressive symptoms would positively correlate to stress induced by negative events in LBC, and that the extent of the difference would vary by the left-behind-related characteristics. Finally, we hypothesized that certain left-behind-related factors would moderate the relationship between depressive symptoms and the type of parental migration.

## Methods

### Design and procedure

This cross-sectional study was based on data from a school-based survey conducted in Chongqing Municipality between December 2012 and June 2013. The study design was approved by the Human Research Ethnics Committee of Third Military Medical University. The approval from officials of sampled schools was obtained in a written form. Parents or legal gardians were contacted to provide consent on behalf of the children to participate in the study.

Thirty-two well trained postgraduates were involved in the data collection as survey administrators. Three or four administrators were responsible for 30–50 children. The survey was carried out in a classroom. Children in junior and senior high schools were allowed to read the questionnaires alone. Pilot tests indicated that they had no problem with reading each item and writing down the answers in an understandable form. During the survey, one or two administrators walked around the classroom to answer participants’ questions and the rest kept the order of the class. Participants were told that there was no right or wrong answer and that they should finish the questionnaires alone and had a right to quit midway at any time. The questionnaires were completed with pencil and paper. To protect children from coercive atmosphere caused by appearance of adult figure, the administrators interacted with them in a very gentle manner. At the beginning of the survey, the administrators explained to participants the right to quit in detail and promised that they would not be punished for quitting midway. To further eliminate participants’ worry about punishment, administrators emphasized again the right to quit during the survey. As a result, 1–2 children quitted midway in most cases.

First, participants were given the following instructions: “Please fill out the personal information form in five minutes”, and then were asked to complete a series of questionnaires designed for estimating the severity of depressive symptoms and impacts of negative life events. After the survey they received notebooks as gifts. The personal information was removed for confidentiality when the data was further analyzed.

### Participants and recruitment

A three-stage cluster survey was conducted in Chongqing Municipality where the LBC phenomenon is prevalent. Research on LBC in Chongqing helps to elucidate the typical features of LBC in China. Chongqing Municipality covers a geographic area of 82,000 km^2^, including 38 counties having a population of 30,170,000. With the assistance of three governmental agencies: Chongqing Office of Poverty Alleviation and Development, Chongqing Foundation for Poverty Alleviation, and Chongqing Municipal Education Commission, we randomly selected 24 counties, with one primary school, one junior and one senior high school in each county, and several classes in each grade.

In the current study, a child was considered as “left-behind” if (1) one or both of his/her parents had migrated to work over the last 6 months or longer; (2) he or she lived with one of his/her parents or other caregivers; (3) he/she was 7–17 years old. LBC were further divided into three subgroups: (1) children left behind by father (LBCF); (2) children left behind by mother (LBCM); (3) children left behind by both parents (LBCB). A child was considered as “control” if he/she had no history of being left behind and lived with both parents at their original residences. Children who were orphans or not 7–17 years old were excluded.

**Fig. 1:**
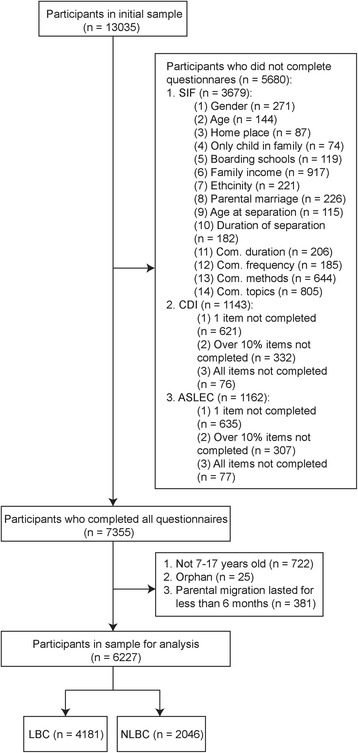
Flow of participants in the study. Note: LBC = left-behind children; NLBC = non-left-behind children; com. = communication between migrant parents and left-behind children; SIF = Socio-demographic Information Form; CDI = Childhood Depression Inventory; ASLEC = Adolescent Self-Rating Life Events Checklist

A total of 13,035 individuals were approached for participation and 6227 of them were included in our analysis. Among the excluded individuals, there were 25 orphans, 30 participants younger than 7 years old, 692 participants older than 17 years old, 381 participants whose parents left for less than 6 months, and 5680 participants who failed to complete all the questionnaire items. The majority of individuals who failed to complete questionnaires did not finish the Socio-demographic Information Form in which items related to family income and communication topics were the mostly missed. As a result, as many as 67.1% (4181/6227) of children had a history of being left behind and the rest 32.9% (2046/6227) without history of being left behind were considered as controls. Figure 1 shows the flow of participants in the study.

### Measures

#### Socio-demographic Information Form (SIF)

The SIF was used to collect information on participants’ age, gender, ethnicity, parental marriage status and family income, and whether participants lodged at schools or had siblings. The frequency, duration, method and topic of communication between migrant parents and children were evaluated as well.

#### Childhood Depression Inventory (CDI)

Children’s depressive symptoms were measured by the Chinese version of CDI, a 27-item self-report questionnaire on five factors: anhedonia (e.g., nothing is fun at all), negative mood (e.g., I am sad all the time), negative self-esteem (e.g., I hate myself), ineffectiveness (e.g., I cannot make up my mind about things) and interpersonal problem (e.g., I do not have any friends) [61]. Participants were asked to recall their feelings and thoughts in the last week and choose options based on their own situations. Each item has three alternatives scored 0, 1, or 2, representing a low, median and high degree of negative self-evaluations, respectively. Half of the items were positively worded and reversely scored. The total score ranges from 0 to 54, and a higher score indicates more serious depressive symptoms. The reliability and validity of the CDI has been verified in participants aged 7–17 years old, and 19 was recommended as the cut-off point for screening of depression [61]. The Chinese version of CDI has been proved to be reliable and valid among Chinese children [14, 16, 62]. For the current study, the internal consistency of the total scale (Cronbach’s α = 0.78) was good, and all factor loads were over 0.30.

#### Adolescent Self-Rating Life Events Checklist (ASLEC)

The degree of negative-event-induced stress was determined by the Chinese version of ASLEC, a 5-point scale consisting of 27 items on six factors: interpersonal conflict (i.e. conflicts between self and others); academic pressure (i.e. failures in exam or heavy course load); punishment (i.e. criticism or corporal punishment); loss (i.e. death of a loved one or loss of property); health and adaptation problems (i.e. severe illnesses and maladjustment to changed diet, daily routine or living environments) and others (i.e. all other types of events) [63]. Participants were asked to report whether the listed events had occurred in the past 3 months and evaluate the effects if any. Each item has six alternatives scored from 0 to 5. If individuals never experienced such negative events, they chose 0. Otherwise, they reported the level of influence ranging from 1 (not at all) to 5 (very much). In order to use two-way ANOVA to explore the interaction effect between negative-event-induced stress and other variables, we transformed the linear slope from a continuous variable into three categories on the basis of a quartile split (bottom, middle two quartiles, and top), with the groups representing the low, median and high level of stress, respectively. The Chinese version of ASLEC has a good reliability (Cronbach’s α = 0.84) and validity (all factor loads are over 0.30) in the Chinese population [63]. The internal consistency of the total scale in present study (Cronbach’s α = 0.77) was good, and all factor loads were over 0.30.

### Statistics

Reliability and internal consistency were estimated using Cronbach’s alpha coefficient. Chi-square analyses were conducted to test between-group differences in left-behind-related and demographic characteristics. MANCOVA, ANCOVA and Bonferroni Post hoc analysis were used to examine the differences in depressive symptoms and negative-event-related stress among NLBC, LBCF, LBCM and LBCB after adjusting for age, ethnicity, home place, parental marriage status, only child in family, lodging at school and family income. Two-way ANOVA and multiple regression analyses were applied to identify the main and interaction effects of NLES, demographic and left-behind-related factors on depressive symptoms. All analyses were conducted using SPSS 21.0. All *p* values were based on two-tailed tests with α = 0.05.

## Results

Table 1 shows the distribution of NLBC and LBC by demographics and family income. The sample consisted of 6227 children aged 7–17 years, with a mean age of 12.68 (Standard Deviation, SD = 2.76). Over 60% of the participants were LBC, which is almost equal to the proportion of samples recruited from the similar rural areas of China in the previous studies [14, 17, 64, 65]. There were more boys than girls in LBCF (53.3% vs. 46.7%), LBCB (51.8% vs. 48.2%) and NLBC (54% vs. 46%), but fewer boys in LBCM (47.5% vs. 52.5%). Most participants were of the ethnic Han origin (over 80%), lived in the countryside (over 75%), had married parents (over 78%) and at least one sibling (over 70%), and approximately half of LBCB lodged at schools (55.2%). Compared to NLBC, a larger proportion of LBC had annual per capita income less than 2300 yuan and a smaller proportion had over 10,000 yuan.

**Table 1:** Demographic and left-behind-related characteristics of the sample (*N* = 6227)

Variables	NLBC	LBC			
	*n*(%)	LBCF	LBCM	LBCB	*k-w χ* ^2b^
		*n*(%)	*n*(%)	*n*(%)	
Total	2046(32.8)	1000(16.1)	202(3.2)	2979(47.9)	
Gender					4.68
Male	1105(54.0)	533(53.3)	96(47.5)	1543(51.8)	
Female	941(46.0)	467(46.7)	106(52.5)	1436(48.2)	
Age (year)					67.35^***^
7–9	364(17.8)	199(19.9)	46(22.8)	379(12.7)	
10–12	700(34.2)	349(34.9)	63(31.2)	870(29.2)	
13–15	532(26.0)	307(30.7)	50(24.8)	1104(37.1)	
16–17	450(22.0)	145(14.5)	43(21.2)	626(21.0)	
Ethnicity					15.58^***^
Han^a^	1782(87.1)	851(85.1)	162(80.2)	2488(83.5)	
Minority	264(12.0)	149(14.9)	40(19.8)	491(16.5)	
Home place					198.73^***^
Small town	510(24.9)	147(14.7)	31(15.3)	303(10.2)	
Countryside	1536(75.1)	853(85.3)	171(84.7)	2676(89.8)	
Parental marriage status					37.45^***^
Married	1869(91.3)	899(89.9)	158(78.2)	2636(88.5)	
Divorced	177(8.7)	101(10.1)	44(21.8)	343(11.5)	
Only child in family	487(23.8)	182(18.2)	54(26.7)	551(18.5)	29.13^***^
Boarding schools	656(32.1)	371(37.1)	78(38.6)	1644(55.2)	290.42^***^
Family income (yuan)^c^					48.58^***^
Less than 2300	400(19.6)	256(25.6)	50(24.8)	764(25.6)	
2300–5000	671(32.8)	353(35.3)	62(30.7)	1035(34.7)	
5000–10,000	511(25.0)	217(21.7)	51(25.2)	672(22.6)	
Over 10,000	464(22.7)	174(17.4)	39(19.3)	508(17.1)	
Age at separation (year)					110.36^***^
0–5		422(42.2)	93(46.0)	1820(61.1)	
6–12		511(51.1)	94(46.5)	1013(34.0)	
13–17		67(6.7)	15(7.4)	146(4.9)	
Duration of separation					2.15
6 months - 2 years		645(64.5)	88(43.6)	1220(41.0)	
2–3 years		135(13.5)	46(22.8)	515(17.3)	
4–5 years		69(6.9)	25(12.4)	374(12.6)	
Over 6 years		151(15.1)	43(21.3)	870(29.2)	
Usual residence (multiple response)					
Father		0(0.0)	79(39.1)	0(0.0)	235.87^***^
Mother		727(72.7)	0(0.0)	0(0.0)	1388.11^***^
Grandparents		381(38.1)	120(59.4)	2297(77.1)	520.00^***^
Siblings		144(14.4)	17(8.4)	418(14.0)	5.34
Other relatives		105(10.5)	24(11.9)	469(15.7)	17.81^***^
Neighbors		32(3.2)	28(13.9)	75(2.5)	2.47
Alone		71(7.1)	17(8.4)	300(10.1)	8.04^*^
Com^d^. frequency					5.17
Daily		144(14.4)	25(12.4)	246(8.3)	
Weekly		519(51.9)	109(54.0)	1734(58.2)	
Monthly		188(18.8)	43(21.3)	551(18.5)	
Half a year		92(9.2)	13(6.4)	248(8.3)	
Over half a year		57(5.7)	12(5.9)	200(6.7)	
Com. duration					71.84^***^
Within 5 min		308(30.8)	54(26.7)	586(19.7)	
5–10 min		332(33.2)	48(23.8)	813(27.3)	
10–30 min		251(25.1)	85(42.1)	1262(42.4)	
Over 30 min		109(10.9)	15(7.4)	318(10.7)	
Com. by telephone		945(94.5)	188(93.1)	2834(95.1)	2.05
Com. by message		67(6.7)	14(6.9)	175(5.9)	1.13
Com. by visiting		33(3.3)	19(9.4)	79(2.7)	28.55^***^
Com. on academic performance		575(57.5)	103(51.0)	1667(56.0)	2.96
Com. on school life		248(24.8)	56(27.7)	860(28.9)	6.17^*^
Com. on life difficulty		124(12.4)	28(13.9)	520(17.5)	14.95^***^
Com. on learning difficulty		108(10.8)	28(13.9)	421(14.1)	7.25^*^
Com. on migrant parental life		225(22.5)	40(19.8)	547(18.4)	32.43^***^
Com. on children’s feelings		299(29.9)	89(44.1)	881(29.6)	18.90^***^

Note: *NLBC* non-left-behind children, *LBC* left-behind children, *LBCF* children left behind by father, *LBCM* children left behind by mother, *LBCB* children left behind by both parents

^*^ *p* < 0.05, ^***^ *p* < 0.001

^a^Han is an ethnic group consisting of the largest population in China

^b^ *k-wxχ* ^2^ value was from the chi-square test

^c^Yuan is a Chinese currency unit

^d^Com. = communication between migrant parents and left-behind children

Table 1 also displays the distribution of LBC based on left-behind-related characteristics. For LBC, being left behind by both parents was the predominant form of separation, followed by being left behind by fathers. Fewest LBC were left behind by mothers. Most children were left behind before 13 years old (93.3% LBCF, 92.5% LBCM and 95.1% LBCB). The children left behind before 6 years old in LBCB (61.1%) were more than those in other subgroups (42.2% LBCF and 46% LBCM). The majority of children were separated from their parent(s) for 6 months to 3 years (78% LBCF, 66.4% LBCM and 58.3% LBCB). LBCM (59.4%) and LBCB (77.1%) mostly lived with their grandparents, but most of the LBCF (72.7%) lived with their mothers. Over half of LBCF, LBCM and LBCB communicated with their migrant parents once a week. LBCF usually communicated with their migrant parents for less than 10 min (64%), but about half of LBCM and LBCB communicated for over 10 min (49.5% LBCM and 53.1% LBCB). Making calls was the most common way of communication (94.5% LBCF, 93.1% LBCM and 95.1% LBCB). Academic performance was the most popular topic (57.5% LBCF, 51% LBCM and 56% LBCB) for parent-child communication in all subgroups, but the proportion of LBCM (44.1%) talking about their feelings than that of other LBC subgroups (29.9% LBCF and 29.6% LBCB).

To verify the hypothesis that LBC may have more depressive symptoms and negative-life-event-induced stress (NLES) than NLBC, we compared depressive symptoms and NLES between NLBC and LBC using MANCOVA after adjusting for age, ethnicity, home place, parental marriage status, only child in family, lodging at school and family income (Table 2). Using Wilks’ statistic, we found a significant effect of left-behind type on depressive symptoms and NLES [Wilks’ λ = 0.98, F (8, 6210) = 13.77, *p* < 0.001]. Following up MANCOVA, we performed univariate ANCOVA to explore the specific group variances of the two variables and the results showed that LBC scored significantly higher on depressive symptoms and NLES than NLBC [F (1, 6217) = 5.93, *p* < 0.05, partial*η* ^2^ = 0.001; F (1, 6217) = 37.26, *p* < 0.001, partial*η* ^2^ = 0.006]. Specifically, LBC scored higher on all types of negative life events involved in the survey than NLBC.

**Table 2:** Comparison between NLBC and LBC regarding depressive symptoms and NLES (*N* = 6227)

Instrumental values	F^a^	Partial *η* ^2^	Mean (SD)	
			NLBC	LBC
			(*n* = 2046)	(*n* = 4181)
Overall MANCOVA Test				
Wilks’ Lambda = 0. 98	13.77^***^	0.17		
Univariate ANCOVA Tests				
Depressive symptoms	5.93^***^	0.001	13.61(6.18)	14.27(6.35)
Total NLES	37.26^***^	0.006	42.14(25.16)	47.28(24.50)
Interpersonal conflict	28.81^***^	0.005	9.80(4.95)	10.17(4.97)
Academic pressure	33.94^***^	0.005	9.36(5.00)	9.92(5.06)
Punishment	17.49^***^	0.003	10.51(7.48)	11.02(7.80)
Loss	10.46^***^	0.002	5.55(4.58)	5.81(4.56)
Health and adaptation problem	84.22^***^	0.013	6.10(4.25)	6.72(4.15)
Other negative events	8.70^***^	0.001	5.05(4.16)	5.26(4.33)

Note: The dependent variables were depressive symptom, total NLES, stress induced by interpersonal conflict, academic pressure, punishment, loss, health and adaptation problem, and other types of negative events

*NLBC* non-left-behind children, *LBC* left-behind rural children, *MANCOVA* multiple analysis of covariance, *ANCOVA* analysis of covariance, *NLES* negative-life-event-induced stress, *SD* standard deviation

^***^ *p* < 0.001

^a^F value was from MANCOVA and ANCOVA results, adjusted for age, ethnicity, home place, parental marriage status, only child in family, studying at boarding school and family income

To examine whether LBC’s depressive symptoms positively correlate to NLES, and whether the extent of the difference varies with the type of parental migration, two-way ANOVA was performed to identify the main effects of NLES and parental migration type and their interaction effects on the depressive symptoms of LBC (Table 3). There was a significant main effect of parental migration type on depressive symptoms [F (2, 4712) = 4.18, *p* < 0.05, *η* ^2^ = 0.002]. The Bonferroni post hoc test revealed that the depressive symptoms were significantly more serious in LBC having migrant parents than those having migrant fathers (*p* < 0.001). NLES also had a main effect on depressive symptoms [F(2, 4712) = 73.11, *p* < 0.001, *η* ^2^ = 0.034]. LBC with higher NLES had more serious depressive symptoms (*ps* < 0.001). However, there was no significant interaction effect between the type of parental migration and NLES on depressive symptoms.

**Table 3:** ANOVAs on depressive symptoms of LBC using type of parental migration and NLES as factors (*N* = 4181)

Independent variables	SS	df	MS	F^a^	*η* ^2^
Type of parental migration^b^ (A)	304.46	2.00	152.23	4.18^*^	0.002
NLES level^c^(B)	5325.04	2.00	2662.52	73.11^***^	0.034
A × B	64.04	4.00	16.01	0.44	0.000
Error	151,939.81	4172.00	36.42		

Note: The dependent variable was depressive symptom

*NLES* negative-life-event-induced stress, *SS* sum of square, *df* degree of freedom, *MS* mean square

^*^ *p* < 0.05, ^***^ *p* < 0.001

^a^F value was from factorial ANOVA results

^b^For the variable parental migration type, 1 = migrant father, 2 = migrant mother, 3 = migrant parents

^c^For the variable NLES, 1 = low, 2 = median, 3 = high

To identify the risk and protective factors of depressive symptoms in LBC, we further conducted hierarchical multiple regression analyses predicting depressive symptoms from NLES and left-behind-related variables. NLES (*b* = 0.09), shorter and less frequent communication (*b* = −0.52, *b* = 0.63) and communication on life difficulties with migrant parents (*b* = 0.65) were associated with more depressive symptoms, whereas communication on academic performance and children’s feelings (*b* = −1.18, *b* = −0.95) linked to fewer depressive symptoms. Compared to LBC having migrant parents, those having migrant fathers had fewer depressive symptoms (*b* = −1.25) (Table 4). These results reveal that NLES, the duration and topic of communication and type of parental migration affect the severity of depressive symptoms of LBC.

**Table 4:** Hierarchical multiple regression analyses predicting depressive symptoms from NLES and left-behind-related variables in LBC (*N* = 4181)

Predictor	ΔR^2^	B(SE)	*β*
Step 1	0.111^***^		
NLES		0.09(0.00)^***^	0.33
Step 2	0.002^**^		
NLES		0.09(0.00)^***^	0.33
Migrant father dummy^a^		−0.72(0.22)^***^	−0.05
Migrant mother dummy^b^		−0.08(0.44)	0.00
Step 3	0.001		
NLES		0.09(0.00)^***^	0.33
Migrant father dummy		−0.81(0.23)^**^	−0.05
Migrant mother dummy		−0.14(0.44)	0.00
Age at separation^d^		0.22(0.16)	0.02
Duration of separation^e^		−0.06(0.08)	−0.01
Step 4	0.031^***^		
NLES		0.09(0.00)^***^	0.31
Migrant father dummy		−1.25(0.55)^***^	−0.08
Migrant mother dummy		−0.50(0.71)	−0.02
Age at separation		0.28(0.16)	0.03
Duration of separation		−0.05(0.08)	−0.01
Com. duration^f^		−0.52 (0.10)^***^	−0.08
Com. frequency^g^		0.63(0.10)^***^	0.09
Com. by telephone dummy^c^		−0.81(0.54)	0.02
Com. by message dummy^c^		0.55(0.50)	0.02
Com. by visiting dummy^c^		0.30(0.37)	0.01
Com. on academic performance dummy^c^		−1.18(0.20)^***^	−0.09
Com. on school life dummy^c^		−0.39(0.20)	−0.03
Com. on learning difficulties dummy^c^		0.05(0.57)	0.00
Com. on life difficulties dummy^c^		0.65(0.27)^**^	0.04
Com. on migrant parental life dummy^c^		−0.34(0.23)	−0.02
Com. on children’s feelings dummy^c^		−0.95(0.20)^***^	−0.07
Adjusted R^2^	0.381^***^		

Note: The dependent variable was depressive symptom

*Com.* communication between migrant parents and left-behind children, *NLES* negative-life-event-induced stress

^**^ *p* < 0.01, ^***^ *p* < 0.001

^a^For the variable migrant father dummy: 0 = migrant parents, 1 = migrant father

^b^For the variable migrant mother dummy: 0 = migrant parents, 1 = migrant mother

^c^For the dummy variables on communication: 0 = no, 1 = yes

^d^For the variable age at separation, 1 = 0–5 years old, 2 = 6–12 years old, 3 = 13–17 years old

^e^For the variable duration of separation, 1 = 6 months – 2 years, 2 = 2–3 years, 3 = 4–5 years, 5 = over 6 years

^f^For the variable communication duration, 1 = within 5 min, 2 = 5–10 min, 3 = 10–30 min, 4 = over 30 min

^g^For the variable communication frequency, 1 = daily, 2 = weekly, 3 = monthly, 4 = half a year, 5 = over half a year

To verify the hypothesis that left-behind-related factors would moderate the relationship between depressive symptoms and NLES, we carried out ANOVAs to illustrate the interaction effects between NLES and left-behind-related factors on depressive symptoms of LBC. Communication duration and frequency, communication by visiting, communication on academic performance, life difficulties and children’s feelings significantly moderated the effects of NLES on depressive symptoms [F (9, 4115) = 4.53, *p* < 0.001, *η* ^2^ = 0.010; F (12, 4115) = 4.89, *p* < 0.001, *η* ^2^ = 0.014; F (3, 4115) = 4.65, *p* < 0.01, *η* ^2^ = 0.003; F (3, 4115) = 12.52, *p* < 0.001, *η* ^2^ = 0.009; F (3, 4115) = 7.80, *p* < 0.001, *η* ^2^ = 0.006; F (3, 4115) = 5.93, *p* < 0.001, *η* ^2^ = 0.004] (Table 5). Therefore, the depressive symptoms of LBC having diverse degrees of stress differed at different levels of left-behind-related variables.

**Table 5:** ANOVAs on depressive symptoms of LBC using NLES and left-behind-related variables as factors (*N* = 4181)

Independent variables	df	MS	F^a^	Partial *η* ^2^
Age at separation^b^ × NLES level (N)	6	40.40	1.15	0.002
Duration of separation^c^ × N	9	70.23	2.00	0.004
Com. duration^d^ × N	9	158.84	4.53^***^	0.010
Com. frequency^e^ × N	12	171.35	4.89^***^	0.014
Com. by telephone^f^ × N	3	53.74	1.53	0.001
Com. by visiting^f^ × N	3	162.82	4.65^**^	0.003
Com. by message^f^ × N	3	16.21	0.46	0.000
Com. on academic performance^f^ × N	3	438.55	12.52^***^	0.009
Com. on school life^f^ × N	3	28.17	0.80	0.001
Com. on life difficulties^f^ × N	3	273.22	7.80^***^	0.006
Com. on learning difficulties^f^ × N	3	67.58	1.93	0.001
Com. on migrant parental life^f^ × N	3	42.18	1.20	0.001
Com. on children’s feelings^f^ × N	3	207.89	5.93^***^	0.004
Error	4115	35.04		

^*^ *p* < 0.05, ^**^ *p* < 0.01, ^***^ *p* < 0.001

^a^F value was from factorial ANOVA results

^b^For the variable age at separation, 1 = 0–5 years old, 2 = 6–12 years old, 3 = 13–17 years old

^c^For the variable duration of separation, 1 = 6 months – 2 years, 2 = 2–3 years, 3 = 4–5 years, 5 = over 6 years

^d^For the variable communication duration, 1 = within 5 min, 2 = 5–10 min, 3 = 10–30 min, 4 = over 30 min

^e^For the variable communication frequency, 1 = daily, 2 = weekly, 3 = monthly, 4 = half a year, 5 = over half a year

^f^For the variables on communication methods and topics, yes = 1 and no = 0

By simple effect analysis (Table 6), we found that at each level of communication duration and frequency, communication by visiting, talking about academic performance, life difficulties and children’s feelings with migrant parents, the depressive symptoms increased as stress increased. For the LBC with a low level of NLES, children having weekly or monthly communication with their migrant parents had significantly fewer depressive symptoms than those having half-yearly communication (*p* < 0.01, *p* < 0.05); communication by visiting and talking about their own feelings with migrant parents significantly reduced LBC’s depressive symptoms (*p* < 0.05, *p* < 0.01). For the LBC with a median level of NLES, children who communicated with migrant parents within 5 min had significantly more depressive symptoms than those who communicated for over 5 min (*p* < 0.05); talking about their own feelings and academic performance with migrant parents also decreased the depressive symptoms (*p* < 0.05, *p* < 0.001), but communication by visiting and talking about life difficulties slightly increased the depressive symptoms (*ps* < 0.05). For the LBC with a high level of NLES, communication on academic performance with migrant parents significantly reduced their depressive symptoms (*p* < 0.05).

**Table 6:** Comparison between depressive symptoms of LBC having a low, median and high level of NLES at different levels of demographic and left-behind-related variables (*N* = 4181)

Variables	NLES			F^a^	Partial *η* ^2^
	Low	Median	High		
	M (SD)	M (SD)	M (SD)		
Com. duration					
(1) Within 5 min	11.99(5.42)	15.77(6.47)	17.57(6.33)	32.72^***^	0.079
(2) 5–10 min	11.41(5.66)	13.94(5.93)	17.27(6.44)	63.07^***^	0.097
(3) 10–30 min	10.65(5.30)	14.09(5.92)	16.06(6.51)	79.88^***^	0.086
(4) Over 30 min	10.83(5.07)	13.84(6.18)	16.31(6.05)	30.15^***^	0.107
F^a^	0.91	3.35^*^	1.35		
Partial *η* ^2^	0.003	0.005	0.004		
Com. frequency					
(1) Daily	10.63(5.24)	13.29(5.83)	14.66(6.15)	14.86^***^	0.060
(2) Weekly	11.03(5.42)	14.04(6.07)	16.57(6.21)	118.22^***^	0.088
(3) Monthly	10.51(5.06)	15.02(6.25)	17.12(6.86)	50.01^***^	0.121
(4) Half a year	13.70(0.78)	15.60(0.49)	17.80(0.59)	9.07^***^	0.056
(5) Over half a year	13.51(1.08)	16.14(0.63)	18.13(0.78)	6.06^***^	0.060
F^a^	3.69^**^	4.32	1.13		
Partial *η* ^2^	0.017	0.009	0.004		
Com. by visiting					
(1) Yes	9.08(4.76)	15.88(6.13)	16.89(6.94)	20.45^***^	0.205
(2) No	11.22(5.28)	14.48(6.07)	16.85(6.53)		
t^b^	−2.40^*^	2.44^*^	0.17		
r	−0.21	0.11	0.01		
Com. on academic performance					
(1) Yes	11.10(5.30)	13.89(5.94)	16.25(6.22)	111.39^***^	0.080
(2) No	11.20(5.63)	15.04(6.26)	17.33(6.64)		
t^b^	−0.26	−4.22^***^	−2.84^**^		
r	−0.01	−0.09	−0.08		
Com. on life difficulties					
(1) Yes	11.26(5.52)	14.50(6.12)	16.88(6.40)	148.67^***^	0.088
(2) No	10.88(5.16)	13.81(5.97)	16.09(6.46)		
t^b^	0.99	2.24^*^	1.75		
r	0.04	0.06	0.06		
Com. on children’s feelings					
(1) Yes	10.47(5.34)	13.98(6.12)	16.33(6.52)	100.52^***^	0.110
(2) No	11.63(5.41)	14.53(6.06)	16.95(6.35)		
t^b^	−3.28^**^	−2.00^*^	−1.60		
r	−0.11	−0.05	−0.05		

Note: The dependent variable was depressive symptom

*NLES* negative-life-event-induced stress, *M* mean, *SD* standard deviation, *Com.* communication between migrant parents and left-behind children

^*^ *p* < 0.05, ^**^ *p* < 0.01,^***^ *p* < 0.001

^a^F value was from ANOVA results

^b^t value was from independent T-test result

Finally, to verify the hypothesis that some left-behind-related variables would moderate the relation between the type of parental migration and depressive symptoms, we performed ANOVAs to examine the interaction effects between the type of parental migration and left-behind-related variables on depressive symptoms. The duration of separation, duration and frequency of communication, communication on academic performance, learning difficulties and children’s feelings had interaction effects with the type of parental migration on depressive symptoms, respectively [F (9, 4117) = 2.06, *p* < 0.05, *η* ^2^ = 0.004; F (9, 4117) = 4.91, *p* < 0.001, *η* ^2^ = 0.011; F (12, 4117) = 5.50, *p* < 0.001, *η* ^2^ = 0.016; F (3, 4117) = 16.55, *p* < 0.001, *η* ^2^ = 0.012; F (3, 4117) = 3.79, *p* < 0.001, *η* ^2^ = 0.003; F (3, 4117) = 9.55, *p* < 0.001, *η* ^2^ = 0.007] (Table 7). This indicates that the depressive symptoms of LBC having migrant father, mother or parents differed at different levels of left-behind-related variables.

**Table 7:** ANOVAs on depressive symptoms of LBC using type of parental migration and left-behind-related variables as factors (*N* = 4181)

Independent variables	df	MS	F^a^	Partial *η* ^2^
Age at separation^b^ × Type of parental migration^c^ (T)	6	80.37	2.10	0.003
Duration of separation^d^ × T	9	78.85	2.06^*^	0.004
Com. duration^e^ × T	9	187.92	4.91^***^	0.011
Com. frequency^f^ × T	12	210.40	5.50^***^	0.016
Com. by telephone^g^ × T	3	26.36	0.69	0.001
Com. by visiting^g^ × T	3	46.96	1.23	0.001
Com. by message^g^ × T	3	74.94	1.96	0.001
Com. on academic performance^g^ × T	3	633.53	16.55^***^	0.012
Com. on school life^g^ × T	3	55.82	1.46	0.001
Com. on life difficulties^g^ × T	1	50.41	1.32	0.000
Com. on learning difficulties^g^ × T	3	145.05	3.79^***^	0.003
Com. on migrant parental life^g^ × T	3	35.85	0.94	0.001
Com. on children’s feelings^g^ × T	3	365.58	9.55^***^	0.007
Error	4117	38.28		

Note: The dependent variable was depressive symptom

*Com.* communication between migrant parents and left-behind children, *MS* mean square

^*^ *p* < 0.05, ^***^ *p* < 0.001

^a^F value was from factorial ANOVA results

^b^For the variable age at separation, 1 = 0–5 years old, 2 = 6–12 years old, 3 = 13–17 years old

^c^For the variable type of parental migration, 1 = migrant father, 2 = migrant mother, 3 = migrant parents

^d^For the variable duration of separation, 1 = 6 months – 2 years, 2 = 2–3 years, 3 = 4–5 years, 5 = over 6 years

^e^For the variable communication duration, 1 = within 5 min, 2 = 5–10 min, 3 = 10–30 min, 4 = over 30 min

^f^For the variable communication frequency, 1 = daily, 2 = weekly, 3 = monthly, 4 = half a year, 5 = over half a year

^g^For the variables on communication methods and topics, yes = 1 and no = 0

By simple effect analysis (Table 8), we discovered that as separating from parents for 6 months to 2 years, LBCF had significantly fewer depressive symptoms than LBCB (*p* < 0.001), but there was no group difference at other levels of separation duration. LBCB had significantly more depressive symptoms than LBCF and LBCM when they communicated with migrant parents for less than 5 min or over 30 min (*ps* < 0.05). LBCB and LBCM had more symptoms than LBCF when communicating for 10 to 30 min (*p* < 0.01, *p* < 0.05). LBCF who communicated with their migrant fathers for over 30 min had significantly fewer symptoms than those communicating for less than 5 min (*p* < 0.05). The LBCB who communicated with their migrant parents for more than 5 min had significantly fewer symptoms than those communicating for less than 5 min (*ps* < 0.05). However, such group differences were not observed among the LBCM. For children having daily communication with their migrant parents, LBCF had significantly fewer depressive symptoms than LBCB (*p* < 0.05). In LBCF and LBCB, those having daily communication with their migrant fathers had significantly fewer symptoms than those having communication over half a year (*ps* < 0.01). LBCF communicating with migrant fathers by visiting had more depressive symptoms than those who did not (*p* < 0.05). LBCF and LBCB talking about academic performance and their own feelings with migrant parents had fewer depressive symptoms than those who did not (*ps* < 0.05).

**Table 8:** Comparison between depressive symptoms of LBCF, LBCM and LBCB at different levels of left-behind-related variables (*N* = 4181)

Independent variables	LBCF (*n* = 1000)	LBCM (*n* = 202)	LBCB (*n* = 2979)	F^a^	Partial *η* ^2^
	Mean (SD)	Mean (SD)	Mean (SD)		
Duration of separation					
(1) 6 months - 2 years	13.20(5.84)	14.32(5.59)	14.55(6.48)	8.32^***^	0.009
(2) 2–3 years	14.38(6.62)	13.93(5.97)	15.32(6.54)	1.49	0.004
(3) 4–5 years	12.70(6.32)	14.48(6.03)	14.44(6.27)	1.97	0.009
(4) Over 6 years	14.36(7.12)	13.81(6.03)	14.16(6.30)	0.18	0.000
F^a^	0.46	1.29	4.29		
Partial *η* ^2^	0.002	0.026	0.004		
Com. duration					
(1) Within 5 min	14.44(6.15)	13.43(5.43)	16.37(6.75)	10.60^***^	0.027
(2) 5–10 min	13.66(6.33)	14.56(6.58)	14.56(6.35)	2.39	0.004
(3) 10–30 min	12.54(6.08)	14.74(5.69)	14.06(6.29)	7.19^**^	0.008
(4) Over 30 min	12.55(5.94)	12.53(4.73)	14.25(6.29)	3.57^*^	0.014
F^a^	3.69^*^	1.73	4.20^**^		
Partial *η* ^2^	0.012	0.035	0.004		
Com. frequency					
(1) Daily	11.63(5.74)	13.60(5.75)	13.33(5.96)	4.49^*^	0.019
(2) Weekly	13.33(6.15)	13.67(5.69)	14.31(6.33)	3.85	0.003
(3) Monthly	13.74(6.47)	14.47(6.11)	14.92(6.74)	2.00	0.005
(4) Half a year	15.08(5.84)	17.31(4.59)	16.24(6.38)	1.37	0.009
(5) Over half a year	16.42(6.01)	15.67(6.31)	16.38(6.54)	0.26	0.003
F^a^	3.58^**^	1.35	3.98^**^		
Partial *η* ^2^	0.007	0.037	0.003		
Com. by visiting					
(1) Yes	15.88(7.52)	13.26(7.05)	14.31(6.41)	1.52	0.020
(2) No	13.42(6.15)	14.28(5.79)	14.54(6.42)		
t^b^	2.25^*^	−0.73	−0.38		
r	0.18	−0.08	−0.02		
Com. on academic performance					
(1) Yes	13.10(5.98)	13.88(6.16)	14.05(6.17)	3.85^*^	0.003
(2) No	14.04 (6.47)	14.49(5.40)	15.40(6.73)		
t^b^	−2.36^*^	−0.75	−5.58^***^		
r	−0.08	−0.05	−0.10		
Com. on learning difficulties					
(1) Yes	13.52(6.40)	14.82(5.27)	14.98(6.56)	1.68	0.005
(2) No	13.50(6.19)	14.08(5.88)	14.44(6.38)		
t^b^	0.04	0.63	1.74		
r	0.00	0.07	0.04		
Com. on children’s feelings					
(1) Yes	12.77(6.01)	12.84(5.46)	14.09(6.55)	5.30^**^	0.006
(2) No	13.81(6.27)	15.24(5.85)	14.86(6.30)		
t^b^	−2.42^*^	−2.98	−3.28^**^		
r	−0.08	−0.21	−0.06		

Note: The dependent variable was depressive symptom

*LBCF* children left behind by father, *LBCM* children left behind by mother, *LBCB* children left behind by both parents. *M* mean, *SD* standard deviation, *Com.* communication between migrant parents and left-behind children

^*^ *p* < 0.05, ^**^ *p* < 0.01, ^***^ *p* < 0.001

^a^ *F* value was from ANOVA results

^b^t value was from independent T-test result

## Discussion

This study has investigated the differences in stress induced by negative life events and depressive symptoms between LBC and NLBC, and how their relationships vary for LBC with different left-behind-related characteristics. Analytical results indicate that LBC are more susceptible to depression than NLBC, and LBCB have more depressive symptoms than LBCF and LBCM, which is consistent with our hypothesis and previous reports [14, 16, 66]. Our results also reveal that more frequent and longer communication with migrant parents could significantly decrease depressive symptoms. Particularly, communications over 5 min correlates to fewer depressive symptoms. Communication on academic performance and children’s feelings relates to fewer symptoms as well. These findings highlight the importance of adequate communication with migrant parents and suitable topics in prevention of LBC’s emotional problems. Given this, we encourage migrant parents to communicate with their left-behind children for more than 5 min each time as frequently as possible, especially on children’s feelings and academic performance.

Our results further show that the level of stress provoked by negative life events is higher in LBC, and the higher level of stress is associated with more depressive symptoms, which verifies our hypothesis and is in line with previous reports [6, 9]. As illustrated in the introduction section, stress is a common factor contributing to depression. However, the relation between stress and depression is complex. For example, studies have shown that (i) the interaction between stressful life events and depressive episode onset is weakened after increasing the number of previous episodes of depression [67] and (ii) reduction in depressive symptoms is associated with a reduction in stress [68]. Fava et al. [68] analyzed the Perceived Stress Scale (PSS) scores of patients with MDD who received treatment with fluoxetine. Although pretreatment scores differed significantly, the post-treatment PSS scores did not differ from those of the control group. They concluded that depression can potentially change cognitive and affective responses to stressors, which might exaggerate the degree of strain and pressure derived from stressful situations. Hence, the higher level of negative-event-related stress reported by LBC may result from more serious depressive symptoms.

In addition, we found communication duration and frequency, communication by visiting, communication on academic performance, life difficulties and children’s feelings moderate the effects of stress on depressive symptoms. Communication duration and frequency affect the depressive symptoms of LBC having low and median levels of NLES, but not those having high levels of NLES. Communication by visiting decreases the depressive symptoms in LBC with low-level stress, but increases symptoms in LBC with median-level stress. Moreover, communication on academic performance reduces depressive symptoms of LBC with median and high levels of stress, and communication on children’s feelings decreases symptoms of those with low and median levels of stress. However, communication on life difficulties augments the severity of depressive symptoms of LBC with median levels of stress. These findings demonstrate that choosing proper topics and methods of parent-child communication according to the levels of stress may be the key to protecting LBC from depression.

Inconsistent with our hypothesis, there is no interaction effect between type of parental migration and stress induced by negative life events on depressive symptoms. However, duration of separation, duration and frequency of communication, communication on academic performance, learning difficulties and children’s feelings respectively moderate the relationship between type of parental migration and depressive symptoms. The significant difference in depressive symptoms between LBCB and LBCF occurs at the early stage of separating from parents (6 months to 2 years), but disappears later, which implies that separation from one parent is as harmful as separation from two parents as long as it lasts for a long time. For the LBC having daily communication with migrant parents or communicating for less than 5 min, 10–30 min or over 30 min, LBCB have more depressive symptoms than other LBC, especially LBCF. This reflects that LBCB is the group most susceptible to depression even if they have adequate communication with parents. LBCF communicating with migrant fathers for less than 5 min have significantly more symptoms than those for more than 30 min, while LBCB communicating for less than 5 min have more symptoms than those for over 5 min. However, LBCM do not have this difference. These findings suggest that compared to LBCB, LBCF probably need more time to communicate with migrant fathers to improve their psychological well-being. In other words, the healing effect of communication may be more difficult to generate in LBCF than in other types of LBC. LBCF have more depressive symptoms when they visit migrant parents than other types of LBC. A possible reason for this result is Chinese fathers’ relatively less emotional expression that amplifies the sense of alienation between themselves and their children. Therefore, migrant fathers should be more patient and skillful as talking with their left-behind children. Furthermore, LBCF and LBCB who talk about academic performance and their own feelings have fewer depressive symptoms than those who do not, which indicates the importance of suitable topics of communication for decreasing depressive symptoms in LBCF and LBCB.

A few limitations with the current research should be noted. First, the study was cross-sectional, only showing correlations rather than causations. Further research should use longitudinal design to record and analyze the relationship between negative life events and depressive symptoms among children before and after being left, thereby exploring the causal role of being left behind. Second, the study only applied self-report questionnaires rather than multiple methodologies such as evaluation by peers, teacher or parents, so that it could not completely avoid social desirability bias or other types of self-defensive responses from subjects. Further refinement of these measures and manipulations is needed. Third, we only focus on relatively simple conditions in which children have different types of parental migration (i.e. migrant father, migrant mother and migrant parents).Yet, other perspectives may exist. For example, we could divide LBC into groups according to their gender or age and then compare the group difference in life events and depressive symptoms. It is warranted for future research to explore other possible combinations. Fourth, the sample sizes for the three subgroups of LBC varied a great deal, ranging from 2979 in LBCB to 202 in LBCM. Such variance may have influenced conclusions about the statistical significance of the results and could be responsible for some nonsignificant findings across these three subgroups. Finally, the effect size of the study was relatively small. We caution readers to keep these sample size differences and small effect size in mind when reflecting on our results.

## Conclusions

Despite the limitations stated above, the current study is one of the first attempts at empirically demonstrating the relationships between negative life events and LBC’s depressive symptoms, highlighting the extent of the difference caused by left-behind-related variables. Our findings reveal a greater risk of depression in LBC compared to NLBC, and children left by both parents are the most susceptible. The NLES is a risk factor of LBC’s depression, but proper and adequate communication between migrant parents and LBC can effectively weaken the link between NLES and depression. However, children left by fathers have more difficulties in improving their psychological health through parent-child communication than children left behind by mothers or both parents. Based on our results, we made several recommendations as follows for the governments, communities, schools and LBC’s families to protect LBC from NLES-related depression.

First, given that long-term separation from parents significantly harms LBC’s psychology, governments should take the responsibility to build regulations helping LBC’s family reunion. For example, they need to amend the existing Hukou system to ensure that LBC can live with migrant parents in cities, without discrimination in education and health insurance. Another feasible method is to encourage employers to provide travel allowance to migrant farmers, so that they are able to go back home to meet children as much as possible. Most importantly, governments should develop local economy of rural areas to attract farmers to work at original residences, thereby fixing the root cause of the LBC problem.

Second, communities having LBC need to establish public welfare organizations protecting LBC’s benefits. These organizations must be agencies for recruiting residents who are willing to take care of LBC (e.g., help to solve life difficulties and provide emotional support) as “surrogate parents”. The “surrogate parents” ought to have skills and knowledge on communication with LBC. For example, they need to know how to choose suitable topics according to the type of parental migration, and to realize the high risk of depression in LBCB and communication difficulties in LBCF. On the other hand, communities should use multiple ways such as poster, seminar and public service advertisement to urge migrant parents to apprehend the negative outcomes of parent-child separation and the benefits of parent-child communication. Meanwhile, the communities should provide migrant parents opportunities of learning skills of communication.

Finally, schools need to set up psychological courses teaching LBC how to regulate emotions, deal with stress and communicate with migrant parents. Furthermore, it is crucial to build psychological counseling centers to help LBC solve mental problems. The schools also need to offer LBC facilities such as telephone and internet for communicating with migrant parents.

## Abbreviations

Com.: Communication
LBC: Left-behind children
LBCB: Children left behind by both parents
LBCF: Children left behind by father
LBCM: Children left behind by mother
MP-LBC: Between migrant parent(s) and left-behind children
NLBC: Non-left-behind children
NLE: Negative life event
